# Inferring Emotions from Speech Prosody: Not So Easy at Age Five

**DOI:** 10.1371/journal.pone.0083657

**Published:** 2013-12-09

**Authors:** Marc Aguert, Virginie Laval, Agnès Lacroix, Sandrine Gil, Ludovic Le Bigot

**Affiliations:** 1 Université de Caen Basse-Normandie, PALM (EA 4649), Caen, France; 2 Université de Poitiers, CeRCA (UMR CNRS 7295), Poitiers, France; 3 Université européenne de Bretagne - Rennes 2, CRP2C (EA 1285), Rennes, France; University of Akron, United States of America

## Abstract

Previous research has suggested that children do not rely on prosody to infer a speaker's emotional state because of biases toward lexical content or situational context. We hypothesized that there are actually no such biases and that young children simply have trouble in using emotional prosody. Sixty children from 5 to 13 years of age had to judge the emotional state of a happy or sad speaker and then to verbally explain their judgment. Lexical content and situational context were devoid of emotional valence. Results showed that prosody alone did not enable the children to infer emotions at age 5, and was still not fully mastered at age 13. Instead, they relied on contextual information despite the fact that this cue had no emotional valence. These results support the hypothesis that prosody is difficult to interpret for young children and that this cue plays only a subordinate role up until adolescence to infer others’ emotions.

## Introduction

The ability to attribute emotions and other mental states to people is an important part of our social life and a crucial skill for children to develop because, unlike physical objects, human behavior is generally interpreted in terms of intentions. Whether adult or child, we understand other peoples’ mental states on the basis of a wide range of cues. For example, one might conclude that I am frightened because of my facial expression or because I have just said that I am afraid or because it is well known that I am always scared before speaking in public. Achim, Guitton, Jackson, Boutin and Monetta [1] suggested a model, the Eight Sources of Information Framework (8-SIF), that describes the cues contributing to what they call mentalization. Some of these cues are immediate, that is gathered from the external world, through the senses, and some of them are stored, i.e. retrieved from memory. Achim and colleagues made another distinction between the cues produced by the agent (the person to whom a mental state is to be attributed) and the cues available in the context surrounding the agent. For example, emotional prosody is a cue providing immediate information about the agent that makes it possible to attribute emotions to this agent. In this study, we investigate the development of the ability to process emotional prosody along with other potentially informative cues. 

Past research has shown that children as young as 5 years are able to attribute an emotional state to a speaker from his/her tone of voice [e.g., 2,3]. However, studies have generally examined this ability using standard emotion-identification tasks in which only one cue is presented. When researchers have tested children in more ecological communication situations in which different kinds of cues are present, as is the case in a real-life environment, the observed performance has not been clear-cut [4,5]. Our study thus aims to further investigate the development of the ability to process emotional prosody along with other potentially informative cues.

It is widely believed that children are very sensitive to prosody as of infancy. Prosody refers to the suprasegmental level of speech. Variations in pitch, intensity, and duration are considered to convey syntactic, emotional and pragmatic information about the utterance and the speaker during spoken interactions [6]. Numerous studies have indeed shown that infants, and even fetuses, have a surprising ability to process prosodic features. In particular, they are able to discriminate their native language from another language and they prefer their mother's voice to that of another woman [7–11]. Moreover, certain studies have investigated the way that infants use prosody to segment the speech stream [e.g., 12].

Surprisingly, the way that infants respond to emotional prosody has been less well investigated [13]. Using a visual habituation procedure, Walker-Andrew and Grolnick [14] showed that 5-month-old children can discriminate between sad and happy vocal expressions. However, this finding was not replicated when no face was present to assist in discrimination [15]. Maestropieri and Turkewitz [16] showed that newborn infants respond in different ways to different vocal expressions of emotion but only in their native language. Whatever the case, the fact that infants discriminate between different acoustic patterns of emotional prosody does not mean that they attribute a mental state to the speaker [17]. 

Prosody-based attribution of emotion has been more fully demonstrated in studies using behavior-regulation tasks in toddlers. Vaish and Striano [18] showed that positive vocalizations without a visual reference encourage 12-month-old children to cross a visual cliff. It should be noted that in this experiment, the infants were cued with both prosody and semantics since their mothers had not been instructed to address meaningless utterances to their children. In the study conducted by Friend [19], 15-month-old infants approached a novel toy more rapidly when the associated paralanguage – prosody and congruent facial expression – was approving rather than disapproving. Similarly, the infants played for longer with a novel toy when the paralanguage was approving than when it was disapproving. However, once again, prosody was not the only cue provided to the children. To our knowledge, only the study by Mumme, Fernald and Herrera [20] permits the conclusion that prosody might be the only factor at work here. Using a novel toy paradigm, these authors showed that fearful emotional prosody conveyed by meaningless utterances was sufficient to elicit appropriate behavior regulation in 12-month-old infants. However, happy emotional prosody did not elicit differential responding.

In older children, standard forced-choice tasks are used. In these, participants have to match prosody with pictures of facial expressions or with verbal labels. Research has shown that children as young as age 4 are able to judge the speaker's emotional state based on prosody at an above-chance level of accuracy [2,3,21–25]. Recent studies have shown that children of about age 5 can use emotional prosody to determine the referent of a novel word [26,27]. Lindström, Lepistö, Makkonen and Kujala [28] showed that school-age children detect emotional prosodic changes pre-attentively. Although young children thus appear to be able to understand some of the meaning conveyed by emotional prosody, this ability increases gradually with age until adulthood [29–32]. Adults are indeed quite efficient at processing prosody in order to attribute basic emotions to a speaker [33]. Moreover, for adults, emotional prosody is a crucial cue in interactions since it primes decisions about facial expressions [34,35] and facilitates the linguistic processing of emotional words [36].

To sum up, the evidence shows not only that the ability to use emotional prosody to attribute an emotional state is present early in life, but also that this ability undergoes a long period of development before reaching the level observed in adults. In addition, the fact that only a limited number of studies have been conducted and that these have varied in their design means that our knowledge about children's abilities is not clear-cut. One problem relates to the number and the type of cues displayed in the studies. For Achim and colleagues [1], the more cues that are available in a mentalizing task the more ecologically valid the task is. Thus in daily interactions, unlike in most experimental forced-choice tasks, prosody is embedded in language and language is embedded in a situation of communication in which many different cues are meaningful. These cues compete with one another and are not always relevant depending on the situation (e.g., prosody is not a relevant cue in computer-mediated communication) and depending on the characteristics of the person who attributes mental states (age, typical/atypical development, etc.) [1]. 

According to the literature, when emotional prosody has to compete with other cues, children preferentially rely on the situational context or on lexical content, to the detriment of emotional prosody, whereas adults prefer the latter. Situational context is defined by three parameters: the participants' location in space and time, their characteristics, and their activities [37]. Aguert et al. [4] showed that when emotional prosody was discrepant with situational context (e.g., a child opening Christmas presents produces an unintelligible utterance with a sad prosody), 5- and 7-year-old children gave precedence to the situational context when judging the speaker's emotional state, while adults relied on prosody, and 9-year-old children used both strategies. Another well-documented finding is that until the age of 9 or 10, children use lexical content to determine the speaker's intention, rather than emotional prosody as adults do [3,22,23,38–40]. For instance, if someone says "It's Christmas time" with a sad prosody, an adult will judge the speaker to be sad, whereas a 6-year-old child will say the speaker is happy. Only specific instructions or priming cause 6-year-olds to rely primarily on emotional prosody [5,41]. Authors refer to these findings as the contextual bias [4] and the lexical bias [3,23], respectively. 

Waxer and Morton [5] argued that there is no specific bias toward lexical content in children. Instead, they suggested that children lack executive control and that this in turn generates cognitive inflexibility and an inability to process multiple cues at the same time. However, this hypothesis was not corroborated. Indeed, the 6-year-old children in their study successfully completed a task involving discrepant lexical and prosodic dimensions of nonemotional speech (e.g., touching an arrow pointing down when they heard the word “high” uttered with a low pitch), whereas they failed in a similar task involving emotional speech (e.g., touching a drawing of a sad face when they heard the word “smile” uttered with a sad prosody). Waxer and Morton therefore finally suggested that "opposite emotion concepts such as happiness and sadness, are strongly and mutually inhibitory" [5]. Resolving the conflict between the two emotions could be very demanding and would require greater mental flexibility. 

Like Waxer and Morton [5], we do not think that there is a specific preference for lexical content. However, unlike these authors, we defend the idea that emotional prosody is a “weak” cue for inferring another person's emotional state and that it is presumably more costly to process than other cues such as lexical content or situational context. Prosody fulfills a wide range of pragmatic and syntactic functions [6,42] and it has been shown that these functions are more or less salient at different points in development [43]. If prosody does not take precedence over other cues in preschool and school-age children, this may simply be because this cue is not prominent at these ages. It would therefore be unnecessary to postulate any cue-specific bias in the understanding of emotions. Two recent studies support the claim that preschoolers are less efficient in recognizing emotional states on the basis of prosody than of other cues. First, Quam and Swingley [24] showed that 2- and 3-year-old children manage to infer a speaker's emotional state (happy or sad) from his or her body language but fail when prosody is the only available cue. The ability to use emotional prosody increases significantly between the ages of 3 and 5 years. Second, Nelson and Russell [32] showed that happiness, sadness, anger and fear were well recognized on the basis of face and body postures, while the recognition of these emotions (except sadness) was significantly poorer in response to prosody in children between 3 and 5 years old. 

In the current study, we asked children to judge the speaker's emotional state on the basis of prosody in situations where the lexical content and the situational context were devoid of emotional valence ("neutral" lexical content and "neutral" situational context). In this way, the emotion conveyed by the prosody was not discrepant with any other cue, whether lexical content or situational context. There was no conflict to resolve that could tax the children's mental flexibility, no mixed emotions, and no opposite emotions that could interfere with each other. If 5-year-old children have trouble inferring the speaker's emotional state from prosody in these conditions (neutral lexical content and situational context), then it would mean that their difficulties are not due to the presence of conflicting emotion cues [5], and this would support the hypothesis that emotional prosody is not a prominent cue for children. In addition, in order to better understand how children infer a speaker's emotional state, we investigated their metacognitive knowledge [44,45]. Metacognitive knowledge involves knowledge about cognition in general, as well as awareness of and knowledge about one’s own cognition. By asking children how they know the speaker’s feelings we should obtain additional information about the cues that led to their choice.

## Methods

### Participants

Eighty French-speaking participants, mainly Caucasian, took part in the experiment. They were divided into four groups on the basis of age: "5-year-olds" (mean age: 5;0, range: 4;10-5;4), "9-year-olds" (mean age: 8;8, age range: 8;7-9;5), "13-year-olds" (mean age: 13;1, range: 12;8-13;6), and "adults" (mean age: 20;11, range: 19;4-28;8). Each group contained 20 participants and had an equal number of females and males. The children were in the normal grade for their age and were attending French state schools that guaranteed a good mix of socioeconomic backgrounds. The adults were university students majoring in psychology. All participants were volunteers and children were a part of experiment with parental consent. The present study was conducted in accordance with the Declaration of Helsinki and approved by the local ethic committee of the laboratory. The experiment was classified as purely behavioral, and the testing involved no discomfort or distress to the participants.

### Material and Procedure

**Figure 1:**
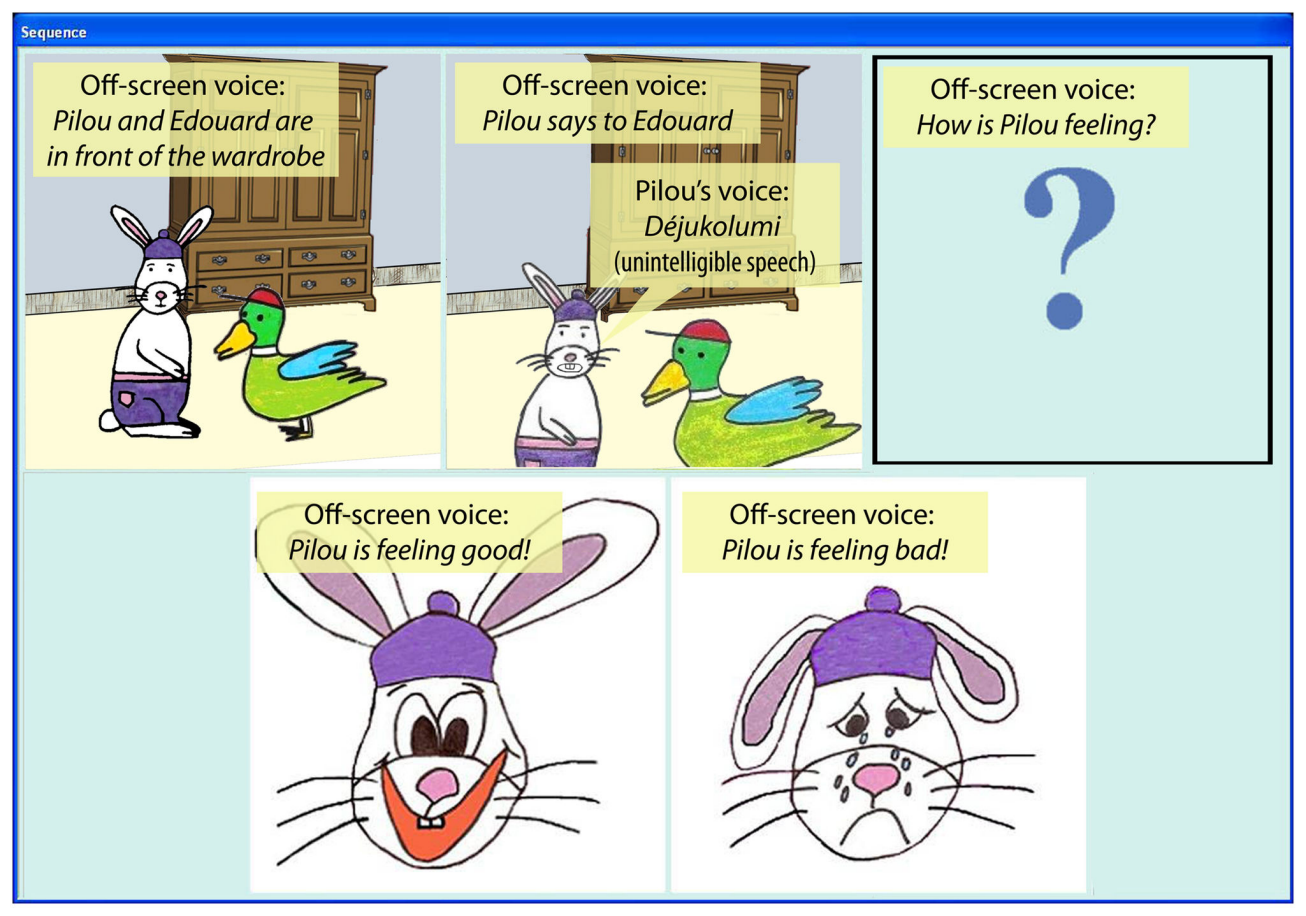
Screen capture of a story just before the participant’s answer (yellow frames have been added to present the audio content of the story).

Six stories containing an emotional utterance produced by one of two story characters were constructed. The stories were computerized using E-Prime software to combine drawings and sounds (see Figure 1). The first drawing provided the setting in which the two characters, i.e. Pilou the bunny and Edouard the duck would interact. When the drawing was displayed, an off-screen narrator's voice was heard describing the situational context. In all the stories, this situational context was neutral. The emotional valence of the situations was pre-tested by a sample of 28 adults, who had to judge the valence of 18 situational contexts on a 5-point scale ranging from 1 (totally negative) to 5 (totally positive). The judges read the description of each situation, which was the same as that which was then spoken by the off-screen narrator in the experiment. Of the 18 situational contexts, 4 were designed to be positive (e.g., “decorating the Christmas Tree”), 4 were designed to be negative (e.g., “being lost in a forest at night”), 10 were designed to be neutral (e.g., “being seated on chairs”). The six most neutral situational contexts – neither negative nor positive – were selected (*m* = 3.08 ; *SD* = 0.09) (see table 1).

**Table 1:** Neutral Situational Contexts.

*Situation*	Pretest Score*
Pilou and Edouard are sitting on chairs	3.00
Pilou and Edouard are in front of a wardrobe	3.00
Pilou and Edouard are in the hall	3.03
Pilou and Edouard are going upstairs	3.07
Pilou and Edouard are looking at each other	3.11
Pilou and Edouard are putting down their glasses	3.25

^*^ Ranging from 1 (very negative) to 5 (very positive). The mean for the 18 situational contexts judged (negative, neutral, and positive) was 3.03.

In the second drawing, the participants saw Pilou talking to Edouard, and heard Pilou's voice producing an utterance. In all stories, the utterance was five syllables long and was purposely made unintelligible – with syllables being randomly mixed – so that the participants could not judge the speaker's emotional state on the basis of lexical content. The participants were told that Pilou spoke a foreign language known only to animals. The emotional prosody employed by the speaker was either positive (happy) or negative (sad). A sample of 14 adults was asked to judge the valence of the prosody of the utterances on a 5-point scale ranging from 1 (very negative) to 5 (very positive). The mean score was 4.84 (SD = 0.08) for the positive-prosody utterances and 1.37 (SD = 0.08) for the negative-prosody utterances. Additionally, a sample of 22 5-year-old children (*M*_*age*_ = 5;0, age range = 4;6–5;5) were asked to judge if the speaker was feeling good or bad after hearing the six utterances without any context. The children gave their answer by choosing between two drawings of Pilou, the speaker. One of the drawings depicted Pilou with a big smile on his face (indicating that he was feeling good) and the other depicted Pilou crying (indicating that he was feeling bad). As in a wide range of studies, this response modality was chosen based on evidence that the ability to associate an emotion with a facial expression is present as of the preschool years [46,47]. Nevertheless, in the familiarization phase, the experimenter carefully explained to the child that the smile meant that Pilou was feeling good and the tears meant that Pilou was feeling bad. No child was excluded because of a lack of understanding. After the children had made their choice, the off-screen voice confirmed the response by saying “Pilou is feeling good” or “Pilou is feeling bad”. We computed the mean number of “feeling good” responses as a function of the valence of the prosody (out of 3). This number was higher when the prosody was positive (*M* = 1.68, *SD* = 0.68) than when the prosody was negative (*M* = 0.86, *SD* = 0.67), *t*(21) = 4.059, *p* < .001. Thus, in each of the six stories, the prosody was emotionally salient, whereas the lexical content was unintelligible and the situational context was neutral. Four additional stories were used as fillers. In the first of these, both the situational context (decorating a Christmas tree) and the prosody were clearly positive. In the second, the context was positive (being on a merry-go-round) and the prosody was neutral. In the third one, both the context (being lost in a forest at night) and the prosody were clearly negative. Lastly, in the fourth story, the context was negative (breaking a toy) and the prosody was neutral.

For each presented story, the child has to evaluate whether the speaker, Pilou, was feeling good or bad (Judgment task). To respond, participants had to choose between the same two drawings as those used in the pretest – Pilou smiling and Pilou crying – by touching one of them on the touch-sensitive screen where they were displayed. The experiment began with a short familiarization phase and two practice stories. Then the stories were presented one by one in random order. After each story, the experimenter asked the following question to the participant: “How do you know that Pilou is feeling good / bad?” and the participant had to explain his/her choice in a few words (Explanation task). Explanations were recorded directly on the computer using dedicated software and a professional microphone.

## Results

### Judgment Task

The dependent variable was the child’s response, which was scored "1" if correct ("Pilou's feeling happy" when the prosody was happy; "Pilou's feeling sad" when the prosody was sad) and "0" otherwise. Analyses of the participants' judgments of the speaker's emotional state were conducted using SPSS software (version 20.0) and a logistic mixed model [see 48,49], with participants as the random intercept, and age, valence, and the age-by-valence interaction as fixed factors. The analyses were conducted on the correct responses. The final model included only significant effects. 

The valence effect and the age-by-valence interaction were not significant. The final model included only one fixed factor since age predicted correct responses, *F*(3, 476) = 16.95, *p* < .001. The odds of correct responses at age 9 were used as the baseline values because the so-called lexical and contextual biases decline around this age [4,23]. As a reminder, odds range from 0 to ∞. If odds = 1, correct and incorrect responses are equally likely. If odds > 1, correct responses are more likely than incorrect responses. If odds < 1, correct responses are less likely than incorrect responses. The odds for giving correct responses (see table 2) were lower in children aged 5 years than children aged 9 (*OR* = .281, CI_95_ [0.156, 0.507], *p* < .001) and were higher in adults than in 9-year-old children (*OR* = 7.628, CI_95_ [2.532, 22.98], *p* < .001). The odds for giving correct responses were not significantly different at 9 and 13 years of age (*OR* = 1.110, CI_95_ [0.578, 2.131], *p* = .753). It should be noted that the five-year-old children's responses were very close to chance (.517). 

**Table 2:** Overall proportions and odds of correct responses as a function of age.

*Age*	*Proportion of correct responses*	*Odds of correct responses*
5-year-old	.517	1.069
9-year-old	.792	3.800
13-year-old	.808	4.217
Adult	.967	29.00

### Explanation Task

 All the explanations given by the participants were transcribed and coded by two independent judges. The overall inter-judge agreement rate was .95. Explanations were sorted into four categories. *Prosody-based explanations* related to the speaker’s prosody or the speaker’s voice. One example of such an explanation was “Because his voice sounded sad”. *Utterance-based explanations* related to the lexical content of the produced utterance. One example was “He said he was glad in a foreign language”. *Context-based explanations* related to the situational context, for example “He likes to be sitting on chairs”. It should be pointed out that sometimes, and especially among the adults, the participant explained his/her choice by referring to the prosody and then immediately explained why the prosody was sad or happy by referring to the context. In this small number of cases (1.2% overall), the explanations were classified as *prosody-based explanations*. Finally, *other explanations* contains the explanations that did not make it clear which cue the participant used to infer the speaker's emotional state: for instance, “He’s feeling bad because he’s bored”. This category also includes cases where no explanation was given (about a third of all cases in the 5-year-olds), explanations that clearly corresponded to a simple paraphrase or repetition of the chosen picture, and irrelevant or unintelligible explanations.

The dependent variable was the child’s explanation, which was scored "1" if based on prosody, “2” if based on context and “3” if the child gave an “other explanation”. Given that utterance-based explanations were quite rare (1.4% overall), these were categorized along with the “other explanations” in order to simplify the interpretation to the results. Analyses of the participants' explanations were conducted using SPSS software (version 20.0) and a multinomial logistic mixed model with participants as the random intercept, and age, response (correct or not), and the age-by-response interaction as fixed factors. The “prosody-based explanations” were chosen as the reference category and 9 years as the baseline age.

**Figure 2:**
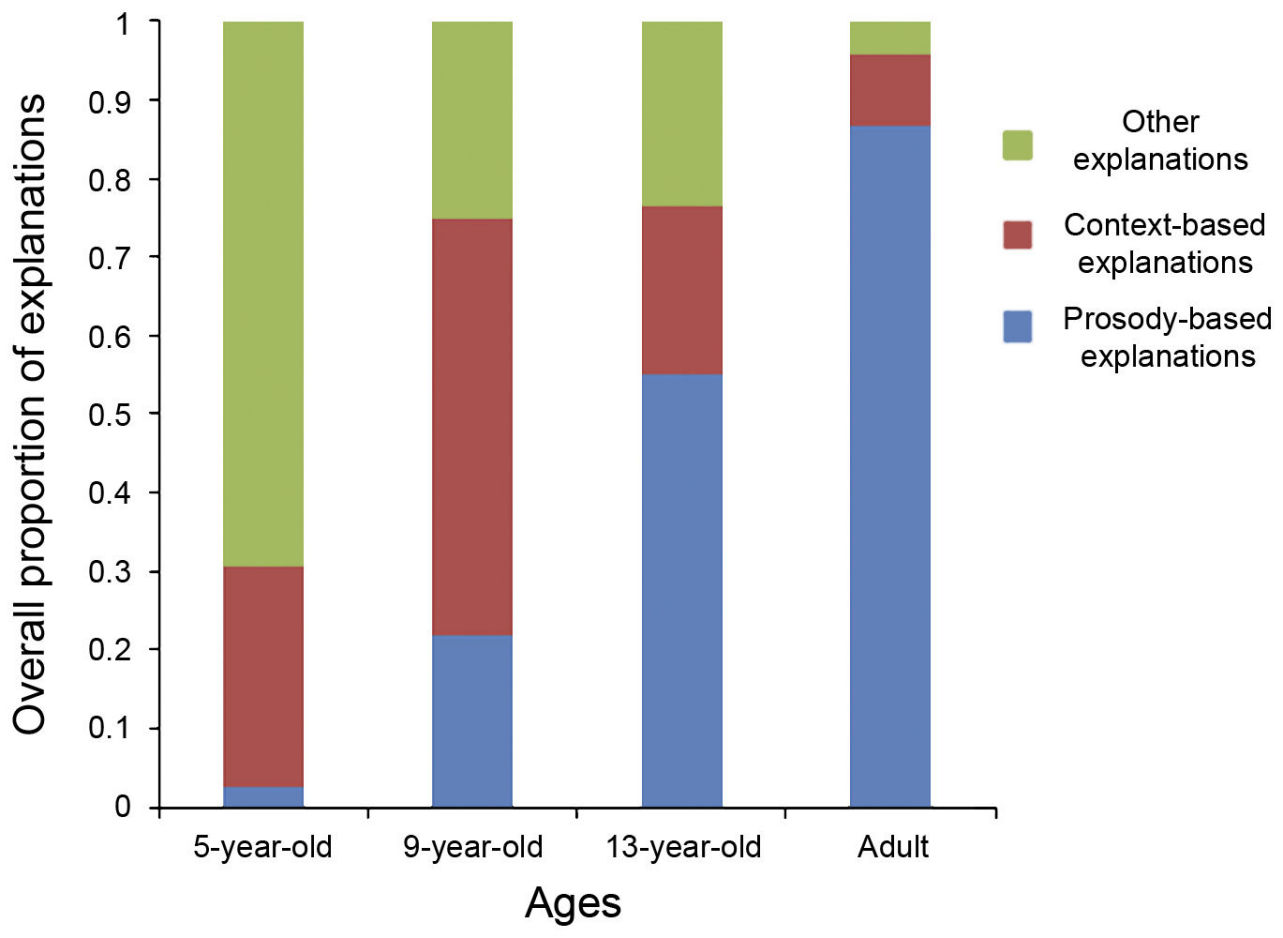
Overall proportions of the different explanations depending on the age.

The response effect and the age-by-response interaction were nonsignificant. The final model included only age, which predicted the type of explanation given by the participants, *F*(6, 472) = 14.22, *p* < .001. An examination of Figure 2 shows that the odds for giving context-based explanations compared to prosody-based explanations were lower in the 13-year-olds and adults than in the 9-year-olds (respectively, *OR* = 0.111 *CI*_95_ [0.030, 0.405], *p* < .001 and *OR* = 0.028, CI_95_ [0.007, 0.112], *p* < .001). There was no significant difference between the 5- and 9-year-olds (*OR* = 2.921, CI_95_ [0.537, 15.896], *p* = .214). In addition, the odds for giving explanations from the category “other explanations” compared to prosody-based explanations were higher in the 5-year-olds and lower in the adults than in the 9-year-olds (respectively, *OR* = 23.47 *CI*_95_ [5.241, 105.1], *p* < .001 and *OR* = 0.038, CI_95_ [0.010, 0.142, *p* < .001). There was no significant difference between the 13- and 9-year-olds (*OR* = 0.364, CI_95_ [0.126, 1.046], *p* = .061).

## Discussion

The aim of this study was to gain a better understanding of how people, and young children in particular, infer the emotional state of a speaker in a multiple-cues environment. More specifically, we investigated the salience of emotional prosody and hypothesized that this cue would be clearly subordinate to other cues, including neutral ones. The participants were asked to judge the emotional state of a speaker and then to explain their judgment.

In line with our hypothesis, the 5-year-old children had difficulty judging the speaker's emotional state on the basis of emotional prosody alone and performed at chance level. The 9- and 13-year-olds did better than the 5-year-olds and were able to infer the speaker's emotional state. However, both the 9- and 13-year-olds were still less accurate than the adults, who performed almost at ceiling level. These results are in line with earlier studies showing that the ability to infer a speaker's emotional state from prosody improves during childhood [21] and until adulthood [29]. Thus, despite the fact that all available cues except emotional prosody were neutral (no cognitive conflict), the youngest children did not take emotional prosody into account. This finding does not support Waxer and Morton's [5] claim that the reason why children fail when two cues constituting two separate information sources are simultaneously available is that their cognitive inflexibility prevents them from taking both cues into account. 

How, then, can we reconcile our results – that the 5-year-old children did not rely on emotional prosody and performed at chance level in the emotion-judgment task – with previous studies showing that children as young as age 3 perform above chance [2,3,21]? The answer may be related to the fact that the utterances used in these other studies were presented completely out of context, whereas our utterances were presented in a neutral context. This is a critical difference, because the simple presence of a context, even a neutral context, may act as a source of information [1]. In line with some studies suggesting that the ability to process information in a holistic manner increases with age [32,50,51], it is conceivable that when faced with various cues, even if some of them are uninformative, children base their judgments on the cue that is the easiest for them to process. A large body of literature has shown that situational context is indeed an important cue for the understanding of other people's emotions in both adults [52] and children [4,53–55].

The analysis of the children’s metacognitive knowledge confirmed that they utilized situational context in order to infer the speaker’s emotional state. *Context-based explanations* were found at each age in childhood irrespective of the responses in the judgment task, i.e. whether they were congruent with the prosody or not. It was not until the age of 13 that the participants gave more *prosody-based explanations* than *context-based explanations*. It seems that 5- and 9-year-old children find it easier to infer the speaker's emotional state from a neutral context – even if this means imagining reasons why the speaker might be happy or sad – than from the speaker's emotional prosody. For instance, when the context was “Pilou and Edouard are going upstairs”, one child said “they are going to their room because they were punished”; another said “because they are going to play”. Morton et al. [41] observed similar behavior in young children (and, to a lesser extent, older children) who attempted to infer emotion from emotionally-neutral lexical content despite the presence of interpretable emotional prosody. In this experiment, we observed few attempts to interpret the lexical content, maybe because the participants had been warned that the speaker spoke a foreign language incomprehensible to humans. 

More data will be needed to explain why 5-year-old children prefer to use a “neutral” situational context rather than a valenced prosody to attribute an emotion to a speaker. However, the existence of this preference has been clearly established by the current study and it leads children to make wrong attributions, or at least, not to make the same inferences as adults. In their model of the sources of information that underpin mentalizing judgments, Achim and colleagues [1] emphasized the issue of cues. They argued that the attribution of mental states depends first and foremost on the ability to choose appropriately between the cues available. They hypothesized that “specific deficits could happen if one or more sources are either under- or overrelied upon relative to the other sources” (p. 124). Our findings confirm Achim et al.’s claims in several ways. First, not all the cues were treated equivalently: the 5-year-olds underrelied on prosody and this impacted on their attributions of mental states. Second, the fact that, in our study, a neutral context was preferred to a valenced prosody indicates that a speaker’s emotional state is inferred more as a function of the nature of the cues than in response to emotional salience. In the model proposed by Achim and colleagues, emotional prosody must be considered as immediate information about the character, while situational context falls into the category of “immediate contextual information”. It is possible that children find it easier to process cues that are extrinsic to the agent than internal cues such as prosody. Third, in line with Achim et al., we stress the importance of using tasks that are as ecological as possible. A neutral-context setting is not equivalent to a no-context setting (as in the emotional prosody pretest). Because the vast majority of interactions in everyday life are contextually situated, this is an important point to consider when examining children’s multi-modal cue processing in general and not just their processing of emotional cues. 

To conclude, our findings show that preschoolers do not primarily rely on prosody when another potential source of information about the speaker’s intent is available, even if that other source is devoid of emotional valence. It seems, then, that there is no cue-specific bias toward lexical content or situational context, but simply that the specific cue in question here – emotional prosody – takes longer to become salient during development [24,29,32]. If this is the case, how can we explain the fact that prosody is the prominent cue in infants but not in preschool children two or three years later? The answer is probably that prosody is prominent in infants due to the lack of competing cues. And this would be even more true for the fetuses. As soon as a new cue makes sense, children use it (e.g., lexical content, see 19). Rather than asking why children do not behave like infants, future research should ask why they do not behave like adults, who clearly rely on prosody to infer a speaker’s emotional state irrespective of the lexical or situational context [3,4] or even to infer the meaning of words [36].
